# Posttranslational Modifications of NPR1: A Single Protein Playing Multiple Roles in Plant Immunity and Physiology

**DOI:** 10.1371/journal.ppat.1005707

**Published:** 2016-08-11

**Authors:** John Withers, Xinnian Dong

**Affiliations:** Howard Hughes Medical Institute-Gordon and Betty Moore Foundation, Department of Biology, Duke University, Durham, North Carolina, United States of America; THE SAINSBURY LABORATORY, UNITED KINGDOM

What makes a protein a good immune regulator? We can make some intuitive guesses based on the characteristics of an immune response. Because immunity is not only dangerous to the pathogen but also potentially harmful to self, its regulators should only be active when the threat of infection is imminent, and their activities should be spatially and temporally appropriate to the infection pressure. In-depth studies of the plant immune regulator non-expressor of pathogenesis-related (PR) genes 1 (NPR1) have revealed the molecular mechanisms contributing to these characteristics and also discovered some surprising connections that the immune system has to normal plant physiological processes to minimize the fitness costs. Because NPR1 transcription is not dramatically affected by pathogen challenge [1], studies of NPR1 have been focused on posttranslational modifications (PTMs). By analogy, NPR1 is like an actor who plays many roles by wearing different costumes.

## What Is the Role of NPR1 in Plant Immune Responses?

The *NPR1* gene was first discovered through genetic screens for *Arabidopsis* mutants compromised in pathogen resistance mediated by the immune signal salicylic acid (SA) [2–8]. SA signaling through NPR1 is required to establish systemic acquired resistance (SAR), an inducible broad-spectrum defense mechanism against secondary infection. Exogenous application of SA is sufficient to bypass the need for an initial infection to induce transcription of the antimicrobial PR genes and enhance general resistance. As SA concentration in the cell increases, NPR1 is translocated into the nucleus and is required for the SA-dependent transcriptomic changes (2,248 out of 2,280 genes in a microarray analysis) [1]. However, because NPR1 does not contain a known DNA binding domain and is not predicted to directly bind DNA, it is hypothesized to be a transcription cofactor.

The conserved role that NPR1 plays as a master immune regulator was demonstrated through overexpression experiments in *Arabidopsis*, rice, and many other plant species [6]. In *Arabidopsis thaliana*, *AtNPR1* confers resistance to an obligate biotroph, *Hyaloperonospora arabidopsidis* Noco and a hemibiotroph *Pseudomonas syringae* pathovar *maculicola* ES 4326 in a dose-dependent manner [5]. In rice (*Oryza sativa*), ectopic expression of *AtNPR1* or overexpression of *OsNH1* (one of the five *AtNPR1* orthologues in rice) [9] leads to increased resistance to rice bacterial blight (*Xanthomonas oryzae* pathovar *oryzae*) and rice blast fungus (*Magnaporthe grisea*) [9–12]. Genome sequence analysis using Basic Local Alignment Search Tool (BLAST) indicates that the *NPR1* gene and amino acid sequences are highly conserved throughout the evolutionary lineage of plants, further illustrating the importance of this master plant immune regulator.

Besides SAR, NPR1 is also required for induced systemic resistance (ISR) in leaves triggered by root-associated rhizobacteria, such as *Pseudomonas fluorescens* WCS417r [13,14]. Different from SAR, ISR resulting from *P*. *fluorescens* colonization is independent of SA accumulation, but requires responsiveness to other plant defense hormones jasmonic acid (JA) and ethylene (Et) [13]. Even though this activity of NPR1 is consistent with its expression pattern and the co-expression of regulatory proteins in roots [15], how NPR1 regulates ISR is not completely understood. In contrast to SAR, which requires nuclear localization of NPR1 [16], ISR requires cytoplasmically localized NPR1 that is thought to be involved in regulation of cross-talk between SA and JA [17–19].

Unlike its positive roles in SAR and ISR, the role that NPR1 plays in local tissue is harder to tease apart due to the entangled interplay between pathogen effectors and the host immune responses at the infection site. The high levels of SA produced at the infection site could enhance basal resistance through NPR1. Moreover, if a pathogen effector or its activity is recognized by a host intracellular nucleotide-binding leucine-rich repeat (NB-LRR) immune receptor, the plant could also mount the effector-triggered immunity (ETI). In contrast to the basal defense mechanisms that promote cell survival against infection, ETI is often associated with programmed cell death (PCD) at the site of infection and is therefore the ultimate immune response to infection by biotrophic and hemi-biotrophic pathogens, which rely on living plant cells for growth [20]. To distinguish the effect of NPR1 on basal defense and ETI, one has to compare infection by the same pathogen with and without the ETI-triggering effector. Such experiments showed that NPR1 is a repressor of ETI and associated PCD [21]. During ETI, NPR1 must be degraded by the proteasome to allow PCD to occur. This degradation is tightly controlled by the SA-mediated differential interactions with the ubiquitin E3 ligase adapters NPR3 and NPR4 so that PCD only occurs at the site of infection [22]. Therefore, NPR1 is a protein that plays a major role in determining life or death of plant cells during ETI.

## What Is the Structure of the NPR1 Protein and Its Molecular Identity?

NPR1 was cloned as a novel protein, and its molecular function and identity were slow to be unveiled. NPR1 consists of an N-terminal bric-a-brac, tramtrack, and broad-complex (BTB) domain, three to four ankyrin repeats, and a C-terminal domain containing a bipartite nuclear localization signal (NLS) and a putative transactivation domain [16,23]. In addition, there are 17 cysteines in *At*NPR1, 10 of which are highly conserved [24], suggesting that the NPR1 protein structure might be redox sensitive. The NLS has been shown to be required for SA-induced NPR1 nuclear translocation and function in SAR. In the nucleus, NPR1 can interact with TGA transcription factors (TFs) through its ankryin repeat domain [25], with NIMIN1 and NIMIN2 TFs through its C-terminal domain, and NIMIN3 by its N terminal BTB domain [26]. Based on transient reporter studies, it was proposed that NPR1 serves as a coactivator of TGA TFs through the putative transactivation domain in the C terminus [23]. Alternatively, like other BTB domain-containing proteins, such as NPR3 and NPR4 [22,27], NPR1 may serve as an adapter for substrate ubiquitination by the Cullin 3 E3 ligase. However, NPR1 has not been found to associate directly with Cullin 3, and a substrate for NPR1-dependent E3 ligase activity has not been firmly identified.

## How Is NPR1 Nuclear Translocation Regulated?

As a transcription cofactor, nuclear localization of NPR1 is required for PR gene expression and the establishment of SAR [16,24]. This process is induced by SA and depends on the NLS present in NPR1. Using an NPR1 fusion to the hormone binding domain (HBD) of the rat glucocorticoid receptor whose nuclear translocation is caused by applying the steroid hormone dexamethasone (DEX), Kinkema et al. demonstrated that DEX treatment alone was not sufficient for induction of *PR-1* expression, indicating that an SA-induced conformational change in NPR1 is also required either for transport of NPR1 into the nucleus or for activation of gene expression [16]. Failed attempts to isolate NPR1 protein from uninduced plants led to the surprising discovery that NPR1 is normally present as a high molecular weight oligomer. Site-directed mutagenesis showed that this oligomer contains intermolecular disulfide bonds between cysteine residues positioned within the BTB domain (Cys^82^) and the region between the BTB and Ankyrin domains (Cys^150^, Cys^155^, Cys^156^, Cys^160^, and Cys^216^) as alanine substitutions at Cys^82^ and Cys^216^ resulted in increased monomer accumulation [24]. In addition, Cys^156^ can be S-nitrosylated to facilitate NPR1 oligomerization in vivo [28]. Upon pathogen infection or accumulation of SA, changes in cellular redox potential lead to the reduction of cysteines through the activity of thioredoxins (TRX-h3 and TRX-h5) and release of NPR1 monomers to localize to the nucleus [28].

NPR1 is not only sensitive to redox changes triggered during plant immune responses but also serves as an intrinsic link between the normal daily redox rhythm and the circadian clock. NPR1 monomer releases peaks at night in phase with the rhythm of SA accumulation [29]. Blocking SA synthesis or NPR1 monomer release significantly dampens the amplitude of the circadian clock, whereas treating plants with SA increases the amplitude of the clock. This allows NPR1 to serve as a redox sensor to modulate the clock activity that gates the different physiological functions (e.g., growth and defense) to minimize conflicts and fitness costs.

Besides modifications of the cysteine residues that affect the NPR1 oligomer–monomer switch, phosphorylation within the C terminal NLS (Ser^589^) by SNF1-related kinase 2.8 (SnRK2.8) was also found to be required for nuclear import and establishment of SAR [30]. Genetic evidence indicates that an additional threonine (Thr^373^), identified by phosphoproteomic analysis of in vitro phosphorylated NPR1, might also be modified by SnRK2.8 as the npr1 (T373A) mutant fails to enter the nucleus and these transgenic lines are deficient in SAR [30].

## What Are the Nuclear PTMs That Regulate NPR1 Functions?

Even though some NPR1 cysteine mutants are constitutively nuclear localized, PR gene expression in these mutants is only moderately elevated and can be further increased by SA treatment [16,24]. This suggests that there may be additional PTMs of NPR1 or NPR1 partner proteins in the nucleus that are SA dependent. NPR1 has been shown to interact with TFs, such as TGAs (typically activators) and NIMINs (typically repressors) by multiple yeast two-hybrid (Y2H) screens [25,26,31]. In yeast, NPR1 can constitutively bind to most of the TGAs (such as TGA3) except TGA1 and TGA4, which contain two redox-sensitive cysteine residues [32,33]. Only the reduced TGA1 and TGA4 are able to interact with NPR1 and induce a reporter when bombarded into *Arabidopsis*. In another example of interplay between NPR1 and TGA TFs, formation of a transcriptionally active NPR1-TGA2 complex requires the core of NPR1s BTB domain (residues 80–91) and oxidation of Cys^521^ and Cys^529^ in the transactivation domain [23].

Despite the strong genetic data supporting a positive role for TGAs in NPR1-mediated PR gene expression, data on a direct physical interaction between NPR1 and TGAs using approaches such as in vitro pull down and super shift in electrophoretic gel mobility shift assays (EMSA) have not been forthcoming. This suggests that a third protein may bridge the interaction between NPR1 and TGA; however, no candidates have been identified. Alternatively, a PTM that is critical for interaction with TGAs may occur to NPR1 in plant nuclei and in yeast used for the Y2H experiment. This modification-dependent interaction might be too transient to be detected in vitro or in EMSA.

Besides TGA TFs, linker scanning mutagenesis of the *PR1* promoter and microarray analyses of SA-mediated gene expression suggest that WRKYs are likely additional TFs, perhaps repressors, of PR genes [1,34]. However, WRKYs have never been identified as NPR1 interactors in the Y2H screens, which discouraged further investigations on their possible interaction using alternative approaches. The recently discovered sumoylation of NPR1 appears to be the missing PTM that reconciles the abovementioned contradictions. Upon SA induction, NPR1 is sumoylated by SUMO3 through the SUMO binding site 3 (SIM3) [35]. The sumoylated NPR1 protein, but not the npr1^sim3^ mutant, can interact with TGA3 in vitro. The opposite is true with regard to NPR1 interaction with WRKY70; while the unmodified NPR1 and the npr1^sim3^ mutant show no interaction with TGA3, both can bind to WRKY70. Chromatin immunoprecipitation experiments found that upon SA induction, there is a shift in NPR1 association from the WRKY-binding W box repressor element to the TGA-binding *as-1* enhancer element in the *PR1* promoter. This shift is abolished in the npr1^sim3^ mutant plants in which npr1^sim3^ is constitutively associated with the W-box at a high level [35]. This failure to switch from association with WRKYs to TGAs may explain the deficiency in PR gene expression and SA-induced resistance of this mutant. It is still unclear, however, whether removal of WRKY repression or activation of TGA or both are important for PR gene induction. If the last scenario is true, then one would expect enrichment of both W-box and the *as-1* element in the promoters of NPR1 target genes. Such a correlation has not been reported.

Since SA-induced sumoylation of NPR1 is potentially the ultimate switch for activating PR genes, it must be tightly regulated. Assuming sumoylation occurs only in the nucleus, NPR1 nuclear translocation could be a limiting factor controlling this modification. Additionally, microarray analysis shows that *SUMO3* expression is induced upon treatment with benzothiadiazole (BTH) (a functional mimic of SA) [1] and, therefore, the availability of SUMO3 is another possible regulatory mechanism. Phosphorylation of NPR1 also affects its interaction with SUMO3 and provides another layer of regulation to fine-tune NPR1 transcription activity. In the absence of SA accumulation, NPR1 is phosphorylated at Ser^55^/Ser^59^, which blocks sumoylation and promotes interaction with WRKY70 to repress *PR1* expression [35]. Furthermore, overexpressing the phospho-mimic npr1^S55/59D^ in *Arabidopsis* blocks the formation of transcriptionally active NPR1-TGA3 complex, which compromises PR gene expression during SA and SAR-inducing treatments and increases susceptibility to virulent *P*. *s*. pv. *maculicola* ES4326 in both locally infected and systemic tissues [35]. In this scenario, Ser^55^/Ser^59^ phosphorylation keeps NPR1 in a stable and inactive state.

**Fig 1:**
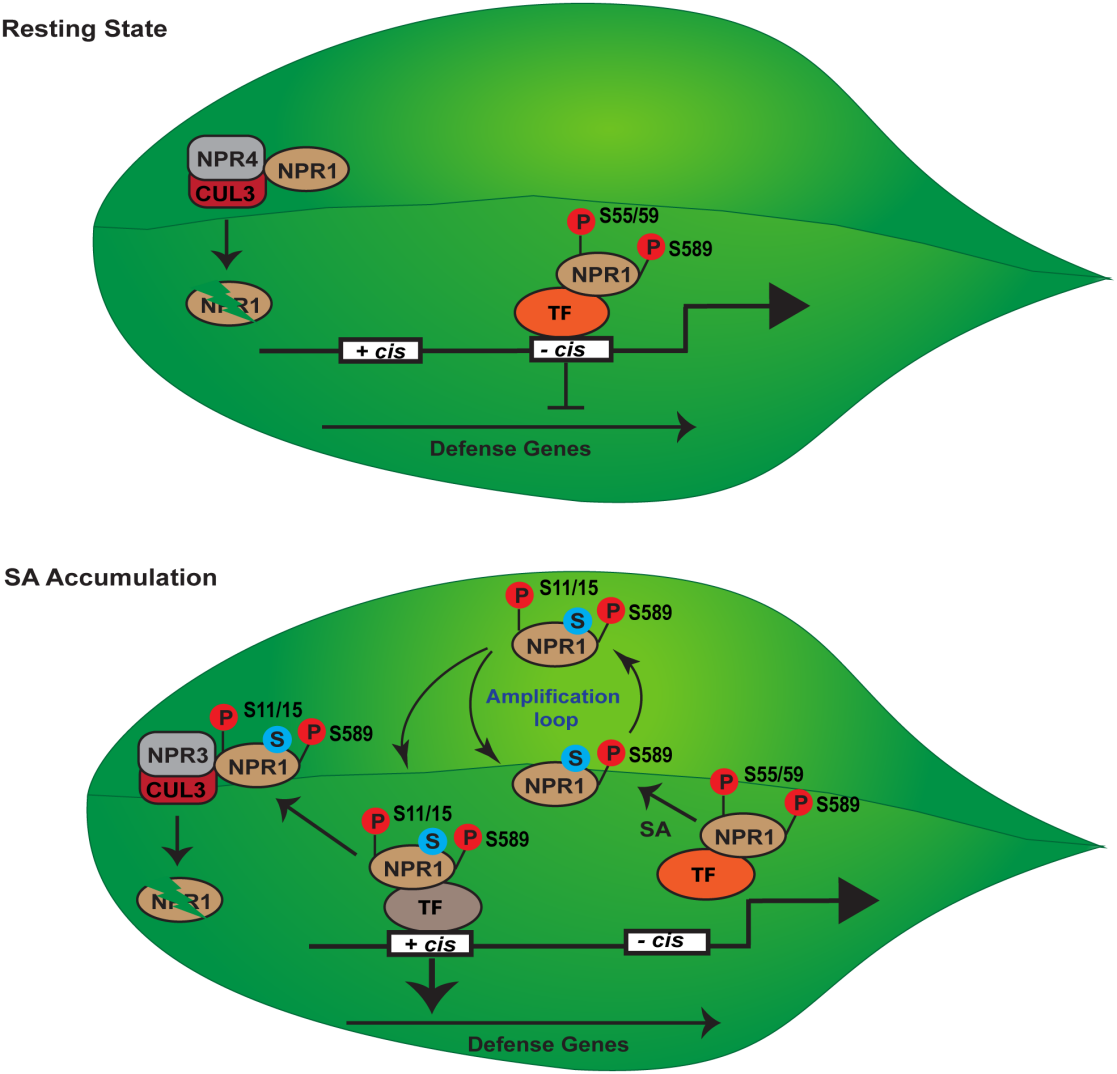
Dynamic regulation of plant immunity through posttranslational modifications of NPR1. Phosphorylation of NPR1 at serine 589 (S589) promotes nuclear localization of NPR1 monomers. At resting state (top panel), NPR1 can be phosphorylated at serine 55 and serine 59 (S55/59), which promotes its association with transcription factors (TF) that bind to repressive cis elements (- cis) in defense gene promoters. NPR1 that is not bound to—cis elements is targeted for degradation through SA-independent interaction with NPR4-CUL3 E3 ubiquitin ligases. As salicylic acid (SA) levels increase (bottom panel), NPR1 is sumoylated (S) and phosphorylated at serine 11 and serine 15 (S11/15), which switches its association with—cis elements to TFs bound to positive cis elements (+ cis) and activates defense genes. Transcriptionally active NPR1 is degraded through SA-dependent interaction with NPR3-CUL3 E3 ubiquitin ligase complexes to ensure full gene induction by refreshing the transcriptional machinery.

Upon induction, NPR1 Ser^55^/Ser^59^ is likely dephosphorylated, allowing NPR1 to become sumoylated. Dephosphorylation of Ser^55^/Ser^59^ and sumoylation of NPR1 are required for a second phosphorylation event at Ser^11^/Ser^15^, which, in turn, further enhances NPR1-SUMO3 interaction to form an amplification loop (Fig 1) [35]. Both the SUMO-deficient (npr1^sim3^) and SUMO-inhibited (npr1^S55/59D^) mutants are unable to be phosphorylated at Ser^11^/Ser^15^. The two phosphorylation events with opposing effects on NPR1 activity and the interplay between phosphorylation, sumoylation, and association with positive and negative TFs may account for the different roles that NPR1 plays during immune responses.

## How Does NPR1 Degradation Regulate Its Activity?

Sumoylation of NPR1 is required for its interaction with not only TGA TFs but also E3 adapter proteins NPR3 and NPR4 (Fig 1) [35]. This ensures that activated NPR1 is also more sensitive to ubiquitination and degradation by the 26S proteasome during SAR. This coupling between activation and degradation renders the transient nature to this immune induction. Consistent with this hypothesis, in the nonphosphorylatable npr1^S55/59A^ mutant in which sumoylation is no longer inhibited by phosphorylation at this site, defense response is constitutively elevated [35]. However, NPR1 degradation was also shown to be required for full activation of defense genes, presumably through refreshing of the transcriptional complexes, as the expression of NPR1 target genes (*WRKY18*, *WRKY38*, *and WRKY62*) was found to be compromised when proteasome activity was chemically inhibited and in the Cullin 3 mutant *cul3a/cul3b* [36]. Therefore, proteasome-mediated degradation of NPR1 makes SAR response stronger but more transient.

Because NPR1 is not only a positive regulator of SAR but also an inhibitor of ETI, it is not difficult to imagine that NPR1 degradation plays a role in ETI induction. Because this PCD-associated immune response is tightly restricted to the site of infection, there is likely a threshold at which NPR1 is degraded to relieve its repression on cell death. Fu et al. proposed that this is achieved by the SA concentration gradient radiating from the site of pathogen challenge [37], combined with differences in the SA binding affinities of NPR3 (low) and NPR4 (high) and the opposing effects of SA binding to their interactions with NPR1 (i.e., SA facilitates NPR3-NPR1 interaction but disrupts NPR4-NPR1 interaction) [22]. NPR1 degradation occurs in cells with high SA concentration (infection site) to remove its inhibition on ETI and PCD. NPR1 is stabilized in cells peripheral to the infection site where SA concentration is too low for NPR3-NPR1 interaction but high enough to disrupt NPR4-NPR1 interaction [22]. Although this is an attractive model for NPR1 regulation of two opposing immune strategies, these dynamic interactions have yet to be demonstrated experimentally. Altogether, NPR1 stability and activity are tightly linked and are regulated by both primary protein structure and PTMs in a manner that provides specificity of the immune response to different challenges.

## What Remains for the Future of NPR1 Research?

Posttranslational modifications of the master immune regulator, NPR1, play a major role in the signaling decision for cell death or cell survival during pathogen challenge. However, there are still some very basic questions that remain to be answered: How is the transcription cofactor activity of NPR1 orchestrated, and what genes does it control during specific immune responses? Modification of NPR1 affects its ability to interact with TFs which may affect their associations with both positive and negative cis*-*elements in the *PR-1* promoter and their activities [35]. Another possibility is that NPR1 may interact with chromatin remodeling proteins. This is consistent with the fact that NPR1 is not only required for transient defense gene expression upon induction but also for the establishment of immune memory long after the inducing signal subsides [38–41]. Since NPR1 is a critical component of signaling during both ETI and SAR, an in-depth analysis of the gene sets that are regulated by NPR1 during each of these immune responses and the modification status of NPR1 at these promoters would shed new light on how plant cells execute specific defense responses.

Although phosphorylation has been shown to regulate multiple NPR1 functions, the specific kinases responsible for some key phosphorylations remain elusive. To date, two members of the SnRK family of kinases have been shown to interact with and phosphorylate NPR1 [30,42], both of which have a positive effect on SA-mediated defense responses. However, the kinases for Ser11/Ser15 and Ser55/Ser59 have not yet been described, and sequence analysis indicates that additional phosphorylation events are likely. Recent studies have suggested that dephosphorylation is likely another regulatory step in NPR1-dependent signaling [35]; however, no phosphatases that directly interact with and regulate NPR1 have been discovered.

Several methods have been used to demonstrate that NPR1 can directly bind SA [43,44]. One suggests that SA binding requires coordination of copper to two cysteines (Cys^521^ and Cys^529^) in the C-terminus and that this activity is crucial for the SA-induced transactivation activity of NPR1 [44]. Given the multiple PTMs that have been shown to regulate NPR1 activity, an investigation of the relationship between NPR1 PTM and SA binding would be quite informative. Structural determination of NPR1 in its modified states and/or bound to SA would provide the ultimate description of the physical dynamics of NPR1 regulation. In spite of two decades of research, additional layers of NPR1 regulation continue to be discovered as we move closer to a complete understanding of this fascinating protein.
